# Developing an eco-bio-social conceptual framework for dengue virus transmission in Latin America and the Caribbean: An e-Delphi study

**DOI:** 10.1371/journal.pgph.0004115

**Published:** 2025-09-16

**Authors:** Aisha Barkhad, Natacha Lecours, Lawrence Mbuagbaw

**Affiliations:** 1 Mary Heersink School of Global Health and Social Medicine, McMaster University, Hamilton, Ontario, Canada; 2 Global Health Division, International Development Research Centre (IDRC), Ottawa, Ontario, Canada; 3 Department of Health Research Methods Evidence and Impact, McMaster University, Hamilton, Canada; 4 Department of Anesthesia, McMaster University, Hamilton, Ontario, Canada; 5 Department of Pediatrics, McMaster University, Hamilton, Ontario, Canada; 6 Biostatistics Unit, Father Sean O’Sullivan Research Centre, St Joseph’s Healthcare, Hamilton, Ontario, Canada; 7 Centre for Development of Best Practices in Health (CDBPH), Yaoundé Central Hospital, Yaoundé, Cameroon; 8 Division of Epidemiology and Biostatistics, Department of Global Health, Stellenbosch University, Cape Town, South Africa; Fundacao Oswaldo Cruz, BRAZIL

## Abstract

Dengue is a rapidly proliferating, mosquito-borne arboviral disease caused by the dengue virus (DENV), and is endemic in Latin America and the Caribbean (LAC). Evidence from the literature suggests that there are several ecological, biological, and social (eco-bio-social) factors determining dengue endemicity in the region. The primary objective of this study was to develop an eco-bio-social conceptual framework for dengue transmission in LAC. The secondary objective was to establish research, and policy and program agendas for dengue prevention and control in LAC. We conducted a sequential, multi-method study using a 3-round e-Delphi method between May and November 2023. Questionnaires were written in English and translated into Spanish and Portuguese, and data were analyzed quasi-anonymously. Professional panelists evaluated the framework using a 10-item, 7-point Likert scale. Consensus was defined as 70% or more agreement among panelists. Panelists also developed a research agenda and evaluated a policy and program agenda using a 12-item, 7-point Likert scale. Eleven dengue professionals from seven LAC countries participated in this study. The most relevant eco-bio-social factors determining dengue transmission were seasonal weather and urban microclimatic oscillations, vectorial capacity and competence, and urbanization and land use. After three consultation rounds, consensus was achieved on a framework comprising 16 ecological, 11 biological, and 28 social factors. Panelists developed a research agenda based on 3 research themes: ecological and environmental; biological and immunological; and social and cultural research. Panelists developed a policy and program agenda for dengue prevention and control, including 4 categories: government investments, integrated programs, intersectoral approaches, and innovative practices. Majority of panelists (88%) agreed that the agenda can improve dengue prevention and control in LAC. The consensus-based eco-bio-social framework and agendas offer novel opportunities to transform dengue prevention and control strategies in LAC and to address the specific needs and experiences of community members in LAC.

## Introduction

Dengue is a proliferating, mosquito-borne arboviral disease caused by the dengue virus (DENV), and affects over 100 countries globally [1]. While a substantial increase in dengue cases has been reported globally over the last years, this increase has been particularly pronounced in the Americas region, where 4.6 million suspected cases and 2 million laboratory-confirmed cases were recorded in 2023 [2]. Dengue has re-emerged in urban centres of Latin America and the Caribbean (LAC) primarily due to the resurgence of the *Aedes aegypti* mosquito vector [3].

The transmission of dengue is determined by an epidemiological system (i.e., episystem) of distinct and interacting ecological, biological, and social (eco-bio-social) factors [4]. Recent evidence indicates that dengue endemicity in LAC is intensified by climate change [5]. The rapid warming of the globe is facilitating *A. aegypti* to proliferate exponentially and exceed current geographical boundaries [6,7]. Increased precipitation influences land cover, including vegetation indices, which can promote *A. aegypti* habitat availability [5,8]. Further, biological factors related to the vector and the virus impact dengue transmission dynamics. For instance, the extrinsic incubation period (EIP) of DENV (i.e., time for DENV to develop inside the mosquito) can vary depending on temperature, thereby influencing the rate and intensity of transmission [9]. Vector capacity and competence determine the mosquito’s ability to acquire, maintain, and transmit DENV, which in turn affects dengue outbreak potential [10]. Additionally, there exist social drivers of DENV transmission including anthropogenic changes to land use [11]; changes to health systems and policies [12]; and changes to the migration of people into urban regions [13]. Urbanization favours the proliferation of *A. aegypti* and improves the range of dengue transmission while making management and control efforts exponentially harder [14].

Dengue also poses a significant political challenge in LAC. A multisectoral response is required at regional and local levels to prevent dengue transmission. However, a fundamental challenge stems from the complexity of the dengue episystem, necessitating a deep understanding of the overall context within which the virus is able to spread. Existing conceptual models for dengue transmission, such as socioecological models [15], and biophysical-ecological frameworks [5], focus on few aspects of disease ecology, often failing to elucidate the dynamic relationships between the eco-bio-social factors involved in the urban dengue episystem.

Ecohealth is a research approach that examines the interactions between humans, animals, and their ecosystems [16]. The first principle of Ecohealth, systems thinking, maintains that the component parts of a system should not be interpreted in isolation. The second principle is transdisciplinary research, which creates new knowledge from dialogue between relevant actors. Therefore, the primary objective of this research was to use a systems thinking Ecohealth approach to develop a visual eco-bio-social conceptual framework that recapitulates the interactions between the factors determining dengue transmission in LAC by consulting with a transdisciplinary panel of dengue researchers and practitioners. The secondary objective was to develop research, and policy and program agendas for dengue prevention and vector control in LAC. The purpose of the framework and agendas is to steer future eco-bio-social dengue research, guide dengue prevention programs, and advise public health policy in LAC.

## Methods

### Ethics statement

To achieve continuous consent, written informed consent was obtained electronically from all panelists in their preferred language at the start of each questionnaire. Confidentiality and anonymity were ensured by using participants’ study codes. Ethical approval was obtained from the Hamilton Integrated Research Ethics Board (HiREB) at McMaster University, Hamilton, Ontario, Canada, to conduct this study (Project Number: 15321).

### Study design

We used a sequential, multi-method study design. We conducted a qualitative scoping review of the literature [17], which informed the subsequent electronic Delphi (e-Delphi) stage (quantitative and qualitative). The e-Delphi method is a recommended tool for synthesizing professional opinion [18], and developing conceptual frameworks [19]. We reported this study according to the ACCORD (ACcurate COnsensus Reporting Document) guidelines [20].

### Population of interest

The target population in this study were English-, Spanish-, French- and Portuguese- speaking researchers and practitioners from LAC with responsibilities and/or technical profiles in entomology, infectious disease epidemiology, sociology, and ecology related to dengue.

e-Delphi panelists were of three primary participant groups:

(i) Researchers actively/previously involved with research on dengue who are/were conducting research on the eco-bio-social determinant(s) of dengue transmission affiliated with LAC institutions.
(ii) Grantees of the World Health Organization’s Special Programme for Research and Training in Tropical Diseases (TDR) project funded by Canada’s International Development Research Centre (IDRC) titled: “*Eco-Bio-Social Approach on Dengue in Latin America and the Caribbean*” (IDRC Project ID: 104951).
(iii) Previous/acting policy and program practitioners involved with dengue interventions or strategic decision-making in governmental or non-governmental capacity.

### Sampling strategy and participant recruitment

The participants were obtained from the target population by employing a non-randomized, purposive sampling strategy using two recruitment methods. First, we recruited authors of papers from the scoping review stage via email. Second, we recruited grantees of the IDRC-funded TDR project via email. We employed the snowballing strategy by asking invited participants to recommend colleagues. The recruitment period for this study began on January 1, 2023, and ended on April 30, 2023.

### Sample size

The e-Delphi panel size relies on group dynamics in reaching an agreement among panelists rather than on statistical power [21]. We aimed for the recommended sample size of 20 panelists [22], since previous literature suggests that this number is sufficient to ensure moderate replicability [23].

### Data collection

Between May 2023 to November 2023, panelists answered online questionnaires on the web-based e-Delphi platform, Welphi (https://www.welphi.com/). Participation was quasi-anonymous, where panelists’ identities were known to the research team but not to one another. This study contained 3 rounds of iteratively developed questionnaires, which were designed in English and translated to Spanish and Portuguese, and then back translated to English to maintain accuracy. Questionnaires were pilot tested in the target languages for comprehensibility with researchers in LAC who were not participants of this study.

After each round, the responses from the questionnaires were aggregated into a report and shared with the panelists. Panelists had the opportunity to adjust their answers and add further comments.

#### First consultation round.

The first consultation round was held between May 2023 to June 2023. Panelists categorized the eco-bio-social factors identified from the scoping review stage as “relevant” or “less relevant”.

Then, panelists provided their feedback on a preliminary eco-bio-social conceptual framework for dengue transmission in LAC developed in the scoping review stage (S1 Fig). The panelists evaluated the preliminary framework using a 10-item and 7-point Likert scale. Likert scales ranged from 1 (strongly disagree) to 7 (strongly agree).

In addition, panelists identified the research gaps that exist for dengue transmission in LAC and began to develop a research agenda. Panelists identified policy and program gaps that exist for dengue prevention and vector control in LAC to develop a policy and program agenda.

#### Second consultation round.

The second consultation round was conducted between July 2023 to August 2023. On a scale of 1–5, the panelists ranked the “importance” and “operability” of the eco-bio-social factors from Round 1 using a concept mapping exercise. The least relevant factor in each category was not included in the exercise. Concept mapping is a structured process designed to organize concepts into categories and generate linkages of specified dimensions [24]. Importance referred to the significance of the factor in determining dengue vector dynamics and transmission relative to the other factors in the same category. Operability referred to the feasibility of potentially acting on the factor through research, policy or programs to improve dengue prevention and control, relative to the other factors in the same category. Importance ranged from 1 (not important at all) to 5 (very important), and operability ranged from 1 (not operable at all) to 5 (very operable). A bivariate graph, divided into four quadrants based on the average of all the importance and operability values in the category was generated for each category of factors [25]. The ‘go-zone’ quadrant (upper right) showed the variables ranked above average for both importance and operability. The ‘no-zone’ quadrant (bottom left) showed the variables ranked below average in both importance and operability indicators.

Then, panelists evaluated the updated eco-bio-social conceptual framework using the same 10-item and 7-point Likert scale as in the previous round and provided additional feedback.

Further, panelists streamlined the research agenda to describe the components (i.e., research themes). Panelists completed a 12-item, 7-point Likert scale to evaluate the policy and program agenda based on 4 criteria: applicability, generalizability, feasibility, and acceptability. Likert scales ranged from 1 (strongly disagree) to 7 (strongly agree).

#### Third consultation round.

The third consultation round was conducted in November 2023. The panelists evaluated the updated eco-bio-social conceptual framework using the same Likert scale as in the previous rounds with the goal of achieving consensus. Panelists identified key audiences that may benefit from the application of the eco-bio-social conceptual framework.

Panelists finalized the research, and policy and program agendas for dengue research, and prevention and vector control. Panelists also described the ways in which the framework could be operated to support the research, and policy and program agenda.

### Data analysis

Data were analyzed quasi-anonymously. We defined consensus as 70% or more of panelists agreeing on all categories of the framework (i.e., percent agreement). We chose 70% as an indication that a substantial number of panelists agreed with what was proposed, while retaining room for dissenting views. There is no consensus as to what is the “best” criterion to use, as these cut-offs are arbitrary, however this threshold is well-established in e-Delphi literature for public health research [19,26–28]. Eq (1) shows the calculation for percent agreement.


$$\%\;agreement=\;\frac{Sum\;of\;number\;of\;``Agree"\;and\;``Strongly\;Agree"\;responses}{Total\;number\;of\;responses}\;\times 100$$ (1)

## Results

### Characteristics of panelists

Out of 50 individuals invited to participate in this study, 11 agreed to participate as panelists. Panelists were from Argentina, Bolivia, Brazil, Colombia, Mexico, Panama, and Uruguay. Two of the panelists were previous grantees of the IDRC-funded TDR project. Some panelists had experience with dengue policy and programming in LAC. All 11 panelists participated in Round 1; 10 participated in Round 2; and 9 participated in Round 3. Panelists’ characteristics are described in Table 1.

**Table 1:** Characteristics of the panelists.

Characteristics	Description	Number of panelists (%)
**Total (n)**		n = 11
**Sex**	Male	7 (63.6%)
	Female	4 (36.4%)
**Country**	Argentina	1 (9.1%)
	Brazil	3 (27.3%)
	Bolivia	1 (9.1%)
	Colombia	1 (9.1%)
	Mexico	2 (18.2%)
	Panama	2 (18.2%)
	Uruguay	1 (9.1%)
**Previous involvement with IDRC-funded TDR project**	Yes	2 (18.2%)
	No	9 (81.8%)
**Years of research experience**	Less than 5	0 (0.0%)
	5-10	3 (27.3%)
	11-15	3 (27.3%)
	More than 15	5 (45.5%)
**Years of dengue policy experience**	Less than 5	5 (45.5%)
	5-10	1 (9.1%)
	11-15	3 (27.3%)
	More than 15	2 (18.2%)
**Years of dengue programming experience**	Less than 5	5 (45.5%)
	5-10	3 (27.3%)
	11-15	3 (27.3%)
	More than 15	0 (0.0%)

### Ranking of relevance

**Fig 1:**
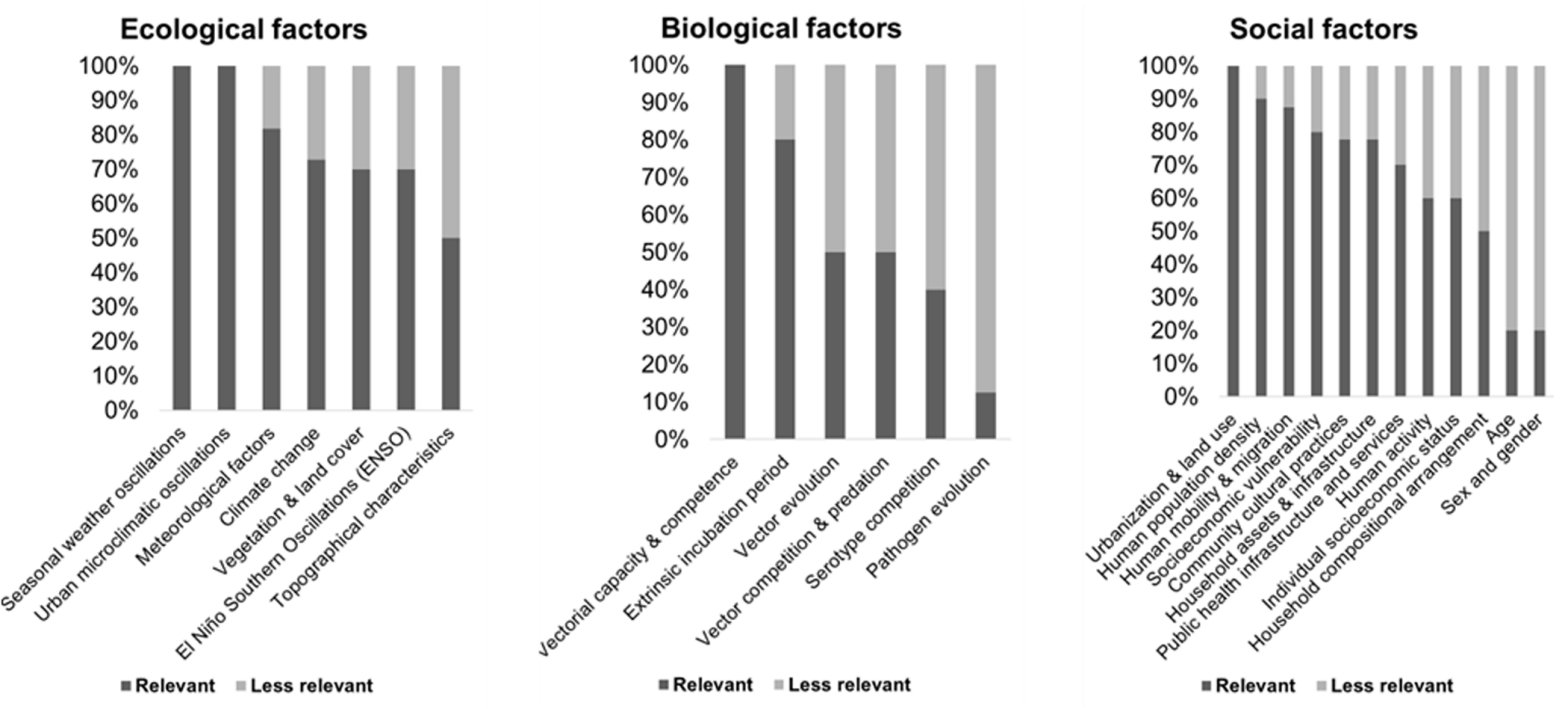
Ranking the relevance of the eco-bio-social factors determining dengue transmission in LAC.

According to panelists, seasonal weather and urban microclimatic oscillations were the most relevant ecological factors in determining dengue transmission. The most relevant biological factor was vectorial capacity and competence. The most relevant social factor was urbanization and land use (Fig 1). The least relevant factors were topographical characteristics, pathogen evolution, age, and sex and gender, and were removed from the subsequent concept mapping exercise.

### Concept mapping exercise

The average importance value for the ecological factors was 4.71 out of 5, and the average operability value was 2.66 out of 5, for 5 ecological factors. On average, panelists viewed that the most important ecological variable was seasonal weather oscillations and meteorological factors, although, this variable was the least operable on average. The only variable in the ‘go-zone’ was climate change.

The average importance value for the biological factors was 3.94 out of 5, and the average operability value was 2.48 out of 5. On average, of the 5 biological factors ranked, panelists viewed that the most important and operable biological variable was the EIP of the virus. The least important variable was serotype competition, and the least operable variable was vectorial capacity and competence.

The average importance value for the social factors was 4.28 out of 5, and the average operability value was 3.83 out of 5. On average, of the 10 social factors, the variables in the ‘go-zone’ were urbanization and land use, human population density, and public health infrastructure and services. The variables in the no-zone were human mobility and migration, community cultural practices, and individual socioeconomic status. The operability average of the social factors was higher compared to the operability averages of the ecological and biological factors.

**Fig 2:**
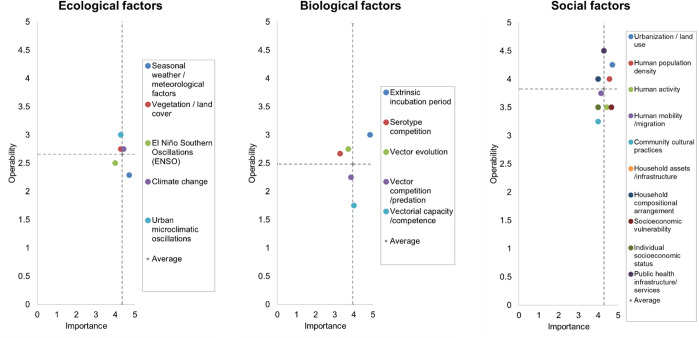
Bivariate ‘go-zone’ plot showing importance and operability rankings for the ecological, biological, and social factors determining dengue transmission in LAC. The quadrants were constructed using the average importance (x) and operability (y) values. The ‘go-zone’ quadrant on the upper right shows the variables that were ranked above average for both importance and operability indicators in the category. The ‘no-zone’ quadrant on the bottom left shows the variables that were ranked below average in both importance and operability indicators in the category. Importance indicator scale: 1 = not important at all; 2 = not important; 3 = somewhat important; 4 = important; 5 = very important. Operability indicator scale: 1 = not operable at all; 2 = not operable; 3 = somewhat operable; 4 = operable; 5 = very operable.

The bivariate plot from the concept mapping exercise is shown in Fig 2.

### Conceptual framework development

**Fig 3:**
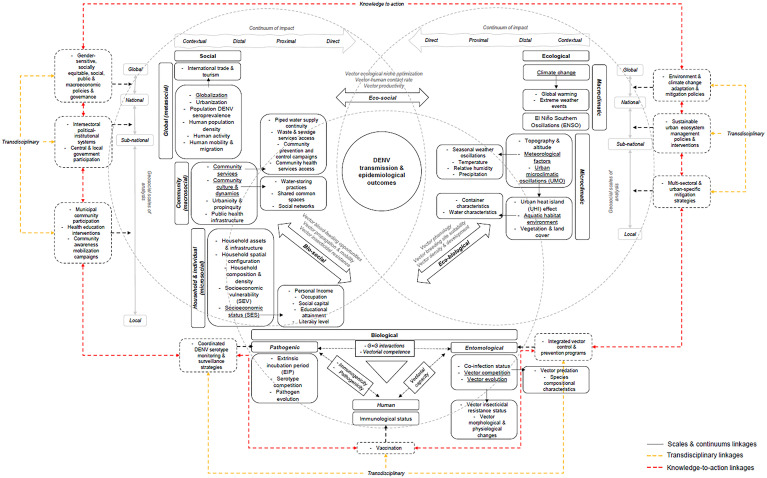
Final visual, comprehensive, eco-bio-social conceptual framework for dengue transmission in LAC. The directivity of the arrows represents the course of action and interaction between variables; dashed arrows represent external and complex pathways of actions and interactions between components; peripheral, political and programmatic factors are signified by dashed boxes; and underlined terms are further expounded in adjacent closed boxes. (G × G – genotype-by-genotype interactions).

Over the 3 consultation rounds, panelists evaluated an eco-bio-social conceptual framework and achieved over 70% agreement (i.e., consensus) on each of the items in a 10-item Likert scale (Table 2). The final framework included 16 ecological factors, 11 biological factors, and 28 social factors (Fig 3).

**Table 2:** Summary of questionnaire items and consensus scores (% agreement) from panelists on an eco-bio-social conceptual framework for dengue transmission in LAC over three consultation rounds^a,b^.

Item	Description	Round 1	Round 2	Round 3
**Item 1.1**	This framework appropriately considers the setting context and timeline from which the data were retrieved from the literature review.	70%	56%	88%
**Item 1.2**	This framework accurately illustrates the ecological factors associated with dengue virus transmission and epidemiological outcomes in the setting context.	80%	78%	88%
**Item 1.3**	This framework accurately illustrates the biological factors associated with dengue virus transmission and epidemiological outcomes in the setting context.	67%	78%	88%
**Item 1.4**	This framework accurately illustrates the social factors associated with dengue virus transmission and epidemiological outcomes in the setting context.	75%	56%	88%
**Item 1.5**	This framework accurately illustrates the external factors in the dashed, peripheral boxes (i.e., policy and program actions) associated with dengue virus transmission and epidemiological outcomes in the setting context.	50%	78%	88%
**Item 1.6**	The relational prepositions of this framework are linked in a cohesive way.	75%	78%	88%
**Item 1.7**	The components of this framework are efficiently integrated.	63%	67%	88%
**Item 1.8**	The geosocial scales of analysis and the continuum of impact are functionally relevant for the interpretation of this framework.	75%	78%	88%
**Item 1.9**	The interpretation of the eco-bio-social conceptual framework is sufficiently substantiated.	88%	78%	88%
**Item 1.10**	The components of this framework consistently reflect a logical translation of diverse perspectives.	75%	78%	88%

^a^Coloured boxes represent non-consensus scores (less than 70% agreement).

^b^7-point Likert scale: strongly disagree; disagree; somewhat disagree; undecided; somewhat agree; agree; strongly agree.

According to panelists, the audiences that may benefit from the application of the conceptual framework include researchers and academics; governmental policy and decision-makers; health institutions, program managers and health authorities; and communities. Table C in S1 Text describes panelists’ views regarding how utilizing the consensus-based eco-bio-social conceptual framework may support the research, and policy and program agendas described below.

### Research agenda development

#### Research gaps.

Research gaps regarding dengue transmission in LAC identified by panelists included: methodological gaps (i.e., gathering data, interpreting data, and using novel research methods); operational and logistical gaps (i.e., accessing resources, estimating and mapping risk, and conducting large-scale studies); and knowledge gaps (i.e., investigating vector and virus surveillance and biological factors, and vaccine development; Table D in S1 Text). All panelists (100%) agreed that the 3 research gaps accurately reflected the current research landscape in LAC.

#### Research agenda.

The research themes in the research agenda developed by panelists included: sociocultural research; entomological, viral, and immunological research; vaccine development research; and epidemiological risk modeling research (Table E in S1 Text). Overall, 89% of panelists agreed that the 4 research themes accurately represented what is needed to address the research gaps.

**Fig 4:**
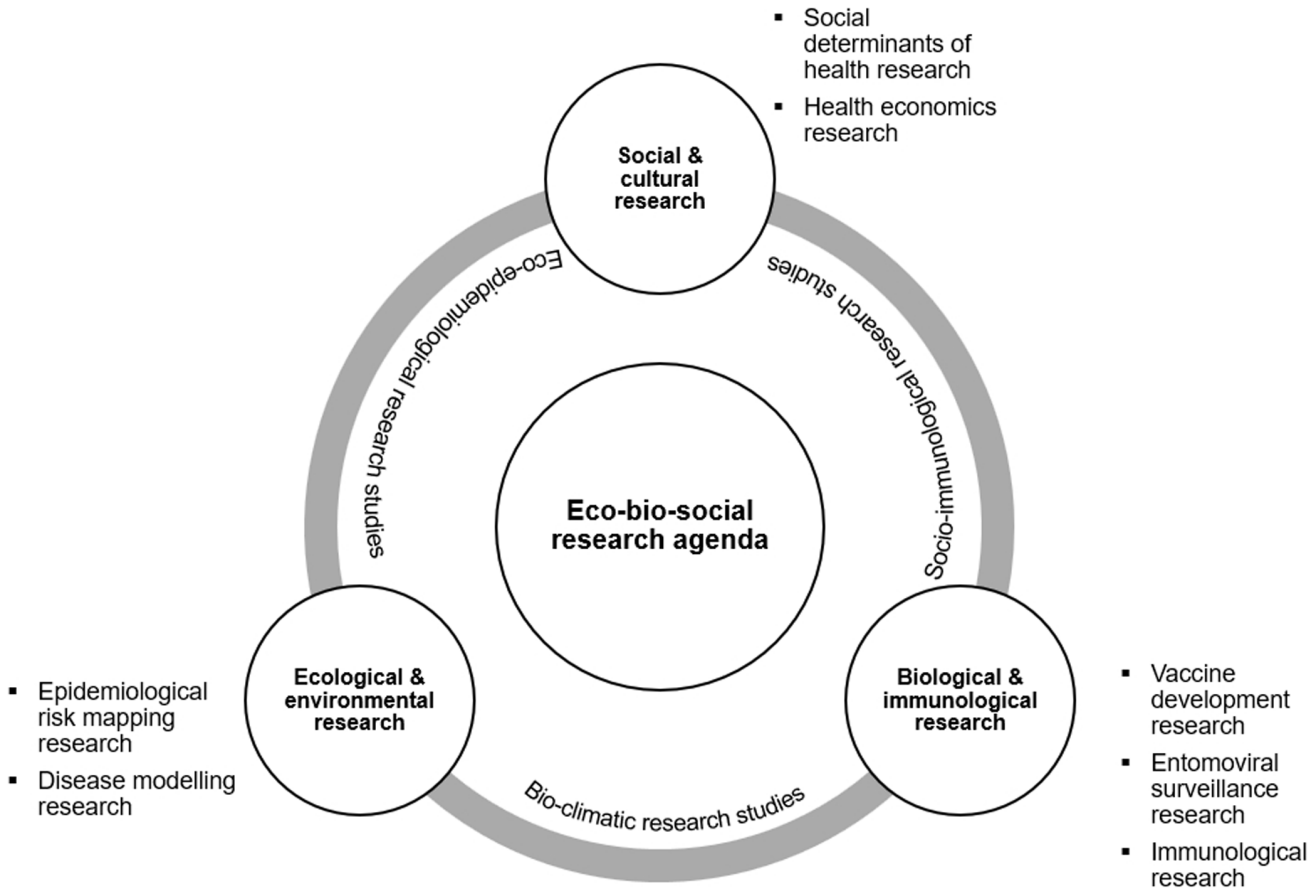
Integrated research agenda for eco-bio-social research on dengue in LAC.

The research gaps and themes contemplated by the panelists were integrated into a research agenda that included 3 research themes: ecological and environmental research; biological and immunological research; and social and cultural research (Fig 4).

### Policy and program agenda development

#### Policy and program gaps.

According to panelists, policy and program gaps that exist for dengue prevention and control in LAC included: diagnostic capacity, digital and synchronized case reporting protocols, homogenous vector surveillance techniques, and monitoring and early warning information systems. All panelists (100%) agreed that the policy and program gaps accurately reflected the status of dengue control and prevention efforts in LAC.

Panelists identified operational constraints that may perpetuate the existence of the policy and program gaps, and included: lack of human resources; lack of logistical and technological resources for diagnosing, detecting, and reporting cases; lack of infrastructural resources; lack of financial resources and government investments; and the presence of urban violence. Panelists also distinguished operational tools which exist to ameliorate policy and program gaps. These tools included: risk stratification to identify dengue hotspots; community-based health education to improve populations’ health literacy; and citizen empowerment.

#### Policy and program agenda.

**Fig 5:**
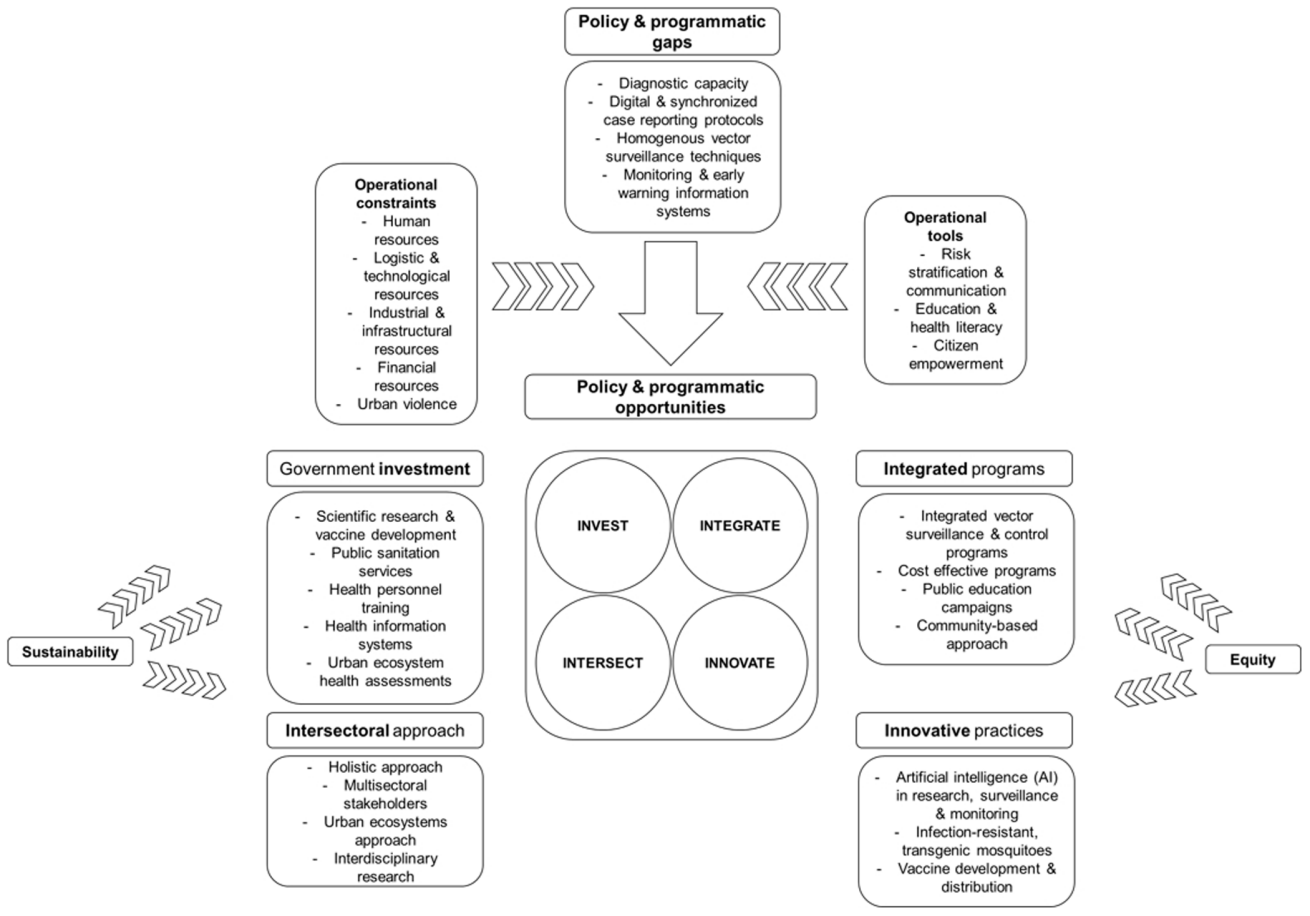
Policy and program agenda for dengue prevention and control in LAC.

The policy and program agenda was categorized into 4 categories, including: government investments, integrated programs, intersectoral approaches, and innovative practices (Fig 5).

Panelists evaluated the policy and program agenda based on 4 criteria: applicability, generalizability, feasibility, and acceptability (Table F in S1 Text; S2 Fig). Overall, 88% of panelists either agreed or strongly agreed that the agenda can improve dengue prevention and control in LAC. Additionally, 44% of the panelists either disagreed or somewhat disagreed that the agenda can be equitably implemented in the region. A group of panelists either disagreed or somewhat disagreed (44%) that the policy and program agenda can be generalizable to many or all countries in LAC. The political feasibility of the agenda was met with a degree of variability among panelists, however most panelists (55%) agreed or strongly agreed that the agenda was politically feasible.

## Discussion

### Conceptual framework for dengue transmission in LAC

While conceptual models for understanding dengue transmission have previously been proposed, existing socioecological and biophysical-ecological frameworks often lack an integrative structure that uses a systems thinking approach to organize diverse components of transmission. Although these frameworks are grounded in empirical evidence, they overlook the perspectives of key stakeholders, limiting their relevance in real-world settings. To integrate the eco-bio-social factors that affect dengue transmission and emergence, Tana *et al.* (2012) adopted a Venn diagram to illustrate factors situated within the episystem [29]. However, this eco-bio-social framework is generic, as described by the authors, and lacks depth and interrogation of how and at which scale the factors impact parameters of dengue transmission dynamics. Our eco-bio-social framework addresses these gaps and provides unique contributions to the literature due to its structured design, integration of socioeconomic, biological, and climatological determinants, and validation through professional consensus, rendering it regionally grounded.

### Utility of the conceptual framework by key audiences

#### For researchers.

Researchers and academics, including scholars from fields such as epidemiology, public health, environmental science, and urban planning, as well as university institutions, can use the framework to facilitate transdisciplinary research collaboration and implement research studies that investigate the complex interactions between the factors determining the transmission of dengue and other arboviruses transmitted by the *Aedes* mosquito.

#### For policy- and decision-makers.

Governmental policy-makers, such as those working in departments and Ministries of health, environment, and social development, as well as local government bodies and public health departments, can apply the framework to inform strategic programming and policy orientation. By considering the multifaceted determinants of dengue transmission, these entities can allocate resources more efficiently, prioritize dengue hotspots for intervention, and integrate health considerations into broader climate and urban policies.

Decision-makers at health institutions, including hospitals, community clinics, and research centers, may use the framework to compartmentalize priority areas by factors. This can inform budget and staffing allocation decisions and the design of effective health promotion campaigns that address prevention and treatment.

#### For program managers.

Program managers of health authorities, such as directors of vector control programs, can use the framework to refine and implement culturally tailored and targeted prevention strategies. Managers with non-governmental organizations (NGOs) and community-based organizations can use the framework to strengthen community advocacy efforts and develop programs that are culturally relevant and community driven.

#### For communities.

Communities in urban centres of LAC can leverage the framework to negotiate their needs and advocate for support from government and non-governmental sectors. Using insights from the framework to recognize how social determinants, such as housing conditions, contribute to disease risk, community members can engage in participatory prevention efforts and call for sustainable infrastructural improvements and public policies.

### Policy recommendations for dengue prevention and vector control in LAC

In our consultations, panelists considered investment domains for dengue prevention and control which included investments in research, vaccine development, and public health infrastructure. Training health personnel to deliver community education through participatory approaches, such as clean-up campaigns, was emphasized to empower community members to take ownership of vector control at home and in their communities [30]. Building resilient monitoring and early warning information systems and urban ecosystem health assessments, which have been useful control strategies implemented by governments in Asia [31,32], were also recommended. Moreover, intersectoral collaboration between government sectors, such as sanitation, urban management, health services, and surveillance was a vital tool highlighted by panelists to develop locally tailored prevention programs.

Panelists mentioned the need for integrated vector surveillance and control programs throughout administrative levels, with cost-effectiveness and ecological sustainability evaluated through community-government partnerships. Although community cultural practices were ranked low on both importance and operability by panelists, possibly due to the observation that cultural behaviors are deeply ingrained and require long-term, trust-based efforts, panelists noted that culturally sensitive community-based approaches integrating entomological surveillance and participation may contribute to better dengue outcomes. Furthermore, innovative practices, such as releasing *Wolbachia*-infected mosquitoes to hinder DENV transmission, were viewed as promising, cost-effective biological control strategies. Given its proven benefits [33–35], expanding cost-effective biological vector control strategies could enhance prevention efforts [36].

Overall, panelists diverged in opinion regarding the feasibility of scaling dengue prevention strategies equitably across LAC. Dengue policies can only exist when they are supported by capable urban infrastructure [37]. However, the enormous investment required for urban infrastructure alone in developing countries poses a significant equity challenge, rendering the adaptability of intersectoral approach unfeasible. Long-term political will and cross-border collaboration in LAC is necessary, especially since the introduction of a widely available vaccine is increasingly likely [38]. Further, sustainability of dengue policies and programs depends not only on political commitments [39], but also on social mobilization and contextually tailored contributions. For instance, although gender was ranked as “less relevant” by panelists, emerging policy perspectives suggest that the consideration of gender norms as a contextual factor in transmission is necessary for achieving equity and, by extension, sustainability [40].

## Study strengths and limitations

Our study has some limitations that should be considered. Firstly, the small sample size (n = 11) may have limited the variety of the collected results. Second, the snowball sampling strategy potentially introduced selection bias, although efforts were made to safeguard representation of panelists and their views, such as allowing participants to adjust responses; having anonymized participation to reduce confirmation bias; and fostering geographic and disciplinary diversity. Third, attrition across consultation rounds may have introduced response bias, due to panelists with dissenting views possibly dropping out [41]. However, the retention rate of participants for this e-Delphi study was over 80%, and literature suggests that 20% to 30% attrition rates can be expected between rounds [42,43]. Finally, it is possible that panelists experienced response fatigue which may have impacted response quality or accuracy, while non-response may have resulted in an overrepresentation of dominant views. To mitigate attrition and response fatigue, we used a user-friendly online platform; sent email reminders; provided multilingual access; and summarized aggregated responses to encourage engagement by validating panelists’ participation.

Our study maintains several strengths. One notable strength is the inclusion of a diverse group of professionals from different LAC countries with varied degrees of dengue burden. This geographically diverse engagement may enhance the perceived relevance and adoption of this study’s findings by health authorities in these countries. In addition, we used an online survey technique, which enabled participants to provide evaluations regardless of their location and addressed logistical and language barriers. Finally, our panel provided transdisciplinary perspectives, ensuring a robust understanding of the complex dengue problem across disciplines.

## Conclusion

Dengue is transmitted in urban centers of LAC through a complex episystem that is not well understood. We operated systems thinking and transdisciplinary research principles of the Ecohealth approach to investigate the relationships between the eco-bio-social factors that establish the urban dengue episystem. Professional panelists achieved consensus on an eco-bio-social conceptual framework which aims to serve as a basis for an operational roadmap that can guide future dengue initiatives in LAC. In addition, panelists generated a research agenda for future dengue research, and a policy and program agenda to inform dengue prevention and control in LAC. These consensus-based agendas represent unique opportunities to supplement existing dengue prevention strategies in LAC.

We recommend that researchers, policy- and decision-makers, and program managers use the framework and agendas to address panelists’ proposed research gaps, investigate the relationships between the factors, and explore the effectiveness of dengue prevention and control policies and programs that address the needs and experiences of communities in LAC.

## Supporting information

S1 Fig Preliminary visual, comprehensive, eco-bio-social conceptual framework for dengue virus transmission in LAC derived from a scoping review of the literature.
(PDF)

S2 Fig 7-point Likert scale chart to evaluate the policy and program agenda.
(PDF)

S1 Table Minimal dataset.
(XLSX)

S1 Text **Table A.** Definitions and criteria for ranking importance and operability. **Table B**. Importance and operability of each eco-bio-social variable. **Table C**. Operating the eco-bio-social conceptual framework to support the research agenda and policy and program agenda. **Table D**. Perceived gaps in the dengue research landscape in LAC according to e-Delphi panelists. **Table E**. Perceived research themes in the dengue research landscape in LAC. **Table F**. Summary of questionnaire items to evaluate the policy and program agenda for dengue prevention and control in LAC.
(DOCX)
