# Supplementary material for: Developing an eco-bio-social conceptual framework for dengue virus transmission in Latin America and the Caribbean: An e-Delphi study
Source: PLOS Glob Public Health. 2025 Sep 16;5(9):e0004115. doi: 10.1371/journal.pgph.0004115 (PMC12440196; doi:10.1371/journal.pgph.0004115)
Supplement: S1 Fig — Preliminary visual, comprehensive, eco-bio-social conceptual framework for dengue virus transmission in LAC derived from a scoping review of the literature. (PDF) [file pgph.0004115.s001.pdf]

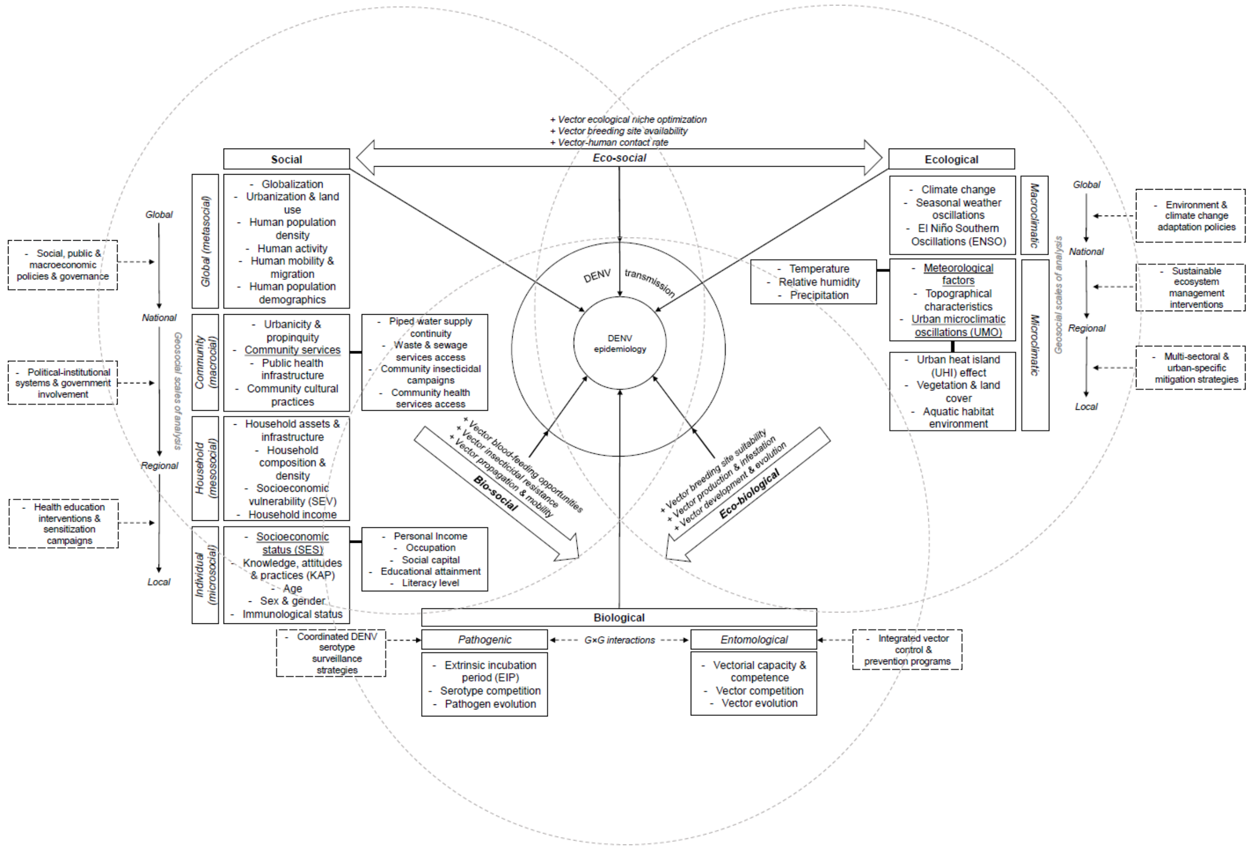


**S1 Fig**. Preliminary visual, comprehensive, eco-bio-social conceptual framework for dengue virus transmission in LAC derived from a scoping review of the literature. (G×G – genotype-by-genotype interactions).
