# Supplementary figures and images for: Developing an eco-bio-social conceptual framework for dengue virus transmission in Latin America and the Caribbean: An e-Delphi study
Source: PLOS Glob Public Health. 2025 Sep 16;5(9):e0004115. doi: 10.1371/journal.pgph.0004115 (PMC12440196; doi:10.1371/journal.pgph.0004115)

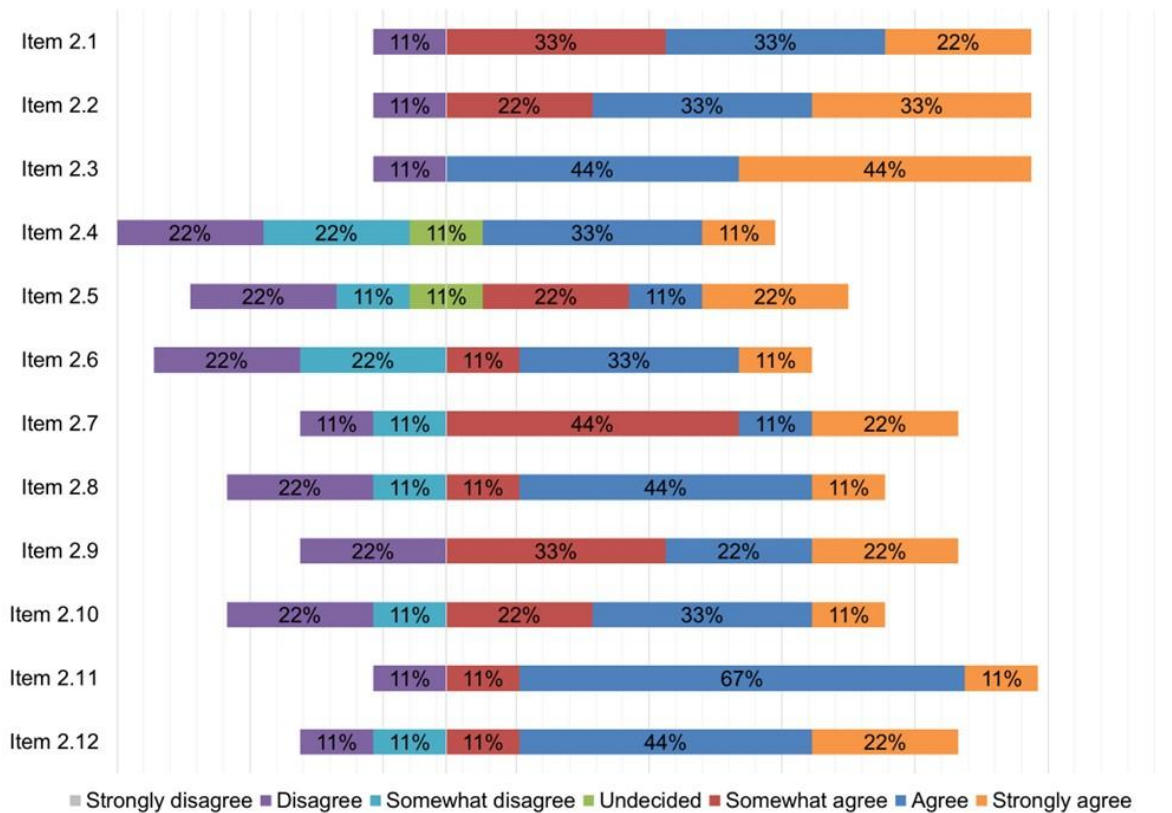

**S2 Fig.** 7-point Likert scale chart to evaluate the policy and program agenda.

Supplement: S2 Fig — 7-point Likert scale chart to evaluate the policy and program agenda. (PDF) [file pgph.0004115.s002.pdf]
