# Supplementary material for: Developing an eco-bio-social conceptual framework for dengue virus transmission in Latin America and the Caribbean: An e-Delphi study
Source: PLOS Glob Public Health. 2025 Sep 16;5(9):e0004115. doi: 10.1371/journal.pgph.0004115 (PMC12440196; doi:10.1371/journal.pgph.0004115)
Supplement: S1 Text — Table A. Definitions and criteria for ranking importance and operability. Table B. Importance and operability of each eco-bio-social variable. Table C. Operating the eco-bio-social conceptual framework to support the research agenda and policy and program agenda. Table D. Perceived gaps in the dengue research landscape in LAC according to e-Delphi panelists. Table E. Perceived research themes in the dengue research landscape in LAC. Table F. Summary of questionnaire items to evaluate the policy and program agenda for dengue prevention and control in LAC. (DOCX) [file pgph.0004115.s004.docx]

**Developing an eco-bio-social conceptual framework for dengue virus transmission in Latin America and the Caribbean: an e-Delphi study**

**Table A**. Definitions and criteria for ranking importance and operability.

| **Ranking score** | **Importance (impact on dengue transmission)** | **Operability (feasibility of intervention)** |
| --- | --- | --- |
| **1 – low** | Minimal impact on dengue transmission | Extremely difficult to intervene or modify |
| **2 – somewhat low** | Indirect or limited influence on dengue transmission | Limited intervention feasibility |
| **3 – moderate** | Some influence on dengue transmission but largely context-dependent | Feasible in specific settings or with targeted interventions |
| **4 – high** | Strong influence on dengue transmission | Generally feasible with appropriate resources |
| **5 – very high** | Critical factor in dengue transmission | Highly feasible with proper coordination and pollical will |

**Table B**. Average importance and operability value for each eco-bio-social variable^a^.

| **Variable** | **Importance average** | **Operability average** |
| --- | --- | --- |
| Seasonal weather oscillations and meteorological factors | 4.714285714 | 2.285714286 |
| Vegetation / land cover | 4.285714286 | 2.75 |
| El Niño Southern Oscillations (ENSO) | 4 | 2.5 |
| Climate change | 4.428571429 | 2.75 |
| Urban microclimatic oscillations | 4.285714286 | 3 |
| Extrinsic incubation period | 4.857142857 | 3 |
| Serotype competition | 3.285714286 | 2.666666667 |
| Vector evolution | 3.714285714 | 2.75 |
| Vector competition /predation | 3.857142857 | 2.25 |
| Vectorial capacity /competence | 4 | 1.75 |
| Urbanization / land use | 4.714285714 | 4.25 |
| Human population density | 4.571428571 | 4 |
| Human activity | 4.428571429 | 3.5 |
| Human mobility /migration | 4.142857143 | 3.75 |
| Community cultural practices | 4 | 3.25 |
| Household assets /infrastructure | 4 | 4 |
| Household compositional arrangement | 4 | 4 |
| Socioeconomic vulnerability | 4.666666667 | 3.5 |
| Individual socioeconomic status | 4 | 3.5 |
| Public health infrastructure/ services | 4.285714286 | 4.5 |

^a^ Importance referred to the significance of the factor in determining dengue vector dynamics, and dengue virus transmission and epidemiological outcomes relative to the other factors in the same category (i.e., to what extent does the factor have an impact on dengue outcomes?).; Operability referred to the feasibility of potentially acting on the factor through research, policy and/or programs in practice to improve dengue prevention and control, relative to the other factors in the same category (i.e., to what extent can research, policies and/or programs address the factor to improve dengue outcomes?).

**Table C.** Operating the eco-bio-social conceptual framework to support the research agenda and policy and program agenda.

|  | **Utility** |
| --- | --- |
| **For research agenda** | **Use a top-down approach** to present the conceptual framework to funding agencies and organizations that decide calls for grants for dengue research in LAC and elsewhere, to foster collaboration, knowledge sharing, and capacity building between networks. |
|  | **Use a bottom-up approach** to promote the framework among research institutions to adopt the framework when programming their research to fill research gaps emphasized by panelists and investigate the separate or integrated aspects of the framework. |
| **For policy and program agenda** | **Streamline the framework** to facilitate the collaboration between researchers, policy- and decision-makers, and program mangers, defining their role in the system and sector, while also enhancing the framework's practicality, usability, and accessibility |
|  | **Involve impactful change agents**, such as the Pan American Health Organization (PAHO), which works closely with the Ministries of Health across the region, that can adopt the framework and ensure policy orientation and program implementation. |

**Table D**. Perceived gaps in the dengue research landscape in LAC according to e-Delphi panelists.

| **Research gaps** | **Specific gaps** | **Description** |
| --- | --- | --- |
| Methodological gaps | Gathering data | There is a perceived paucity of data being collected on vector density at fine scales, both temporally and spatially. |
|  | Interpreting data | Due to the effect of the scales of analysis (i.e., global, national, sub-national, local), there is considerable complexity regarding the analyses of the relationships among climate, environment, social structure, and dengue transmission. This presents a significant research gap. The challenges of adequate statistical models, collecting qualified data, and correctly interpreting analysis models can impose severe restrictions on research and empirical studies. |
|  | Using novel research methods | There is a need to leverage artificial intelligence (AI) and the machine learning models as a novel research method that may predict possible epidemics and aid with the collection of diagnostic and surveillance data. |
| Operational and logistic gaps | Accessing resources | There is a perceived lack of economic and human resources to carry out proper monitoring at the programmatic level, and thus, research is equally lacking. Moreover, the funding for eco-bio-social research on dengue is scarce in LAC. |
|  | Estimating and mapping risk | Research is needed on the transmission networks of DENV between localities. Communication and transmission networks should be established within localities that can be receivers, amplifiers, repeaters, and disseminators while there are other localities that can be captive receivers of the transmission but not participate as active centers of dissemination. This is why risk stratification, estimation, and mapping within DENV epidemic areas and hotspots needs to be improved to enhance control. |
|  | Conducting large-scale studies | Large-scale studies are needed on the surveillance of DENV cases and circulating serotypes. Global scale understanding of the movement of humans and materials and the impacts on dengue transmission are also needed. |
| Knowledge gaps | Investigating vector and virus surveillance | There is a need to assess the distribution of vector species other than *Ae. aegypti* (e.g., *Ae. albopictus*) and the surveillance and distribution of distinct DENV serotypes. |
|  | Investigating biological factors | There is a need for more studies on the biological factors determining vector distribution and DENV transmission. Experiments on vectorial capacity and resistance status are needed. This also includes research on Wolbachia-parasitized mosquitoes. Additionally, studies are needed on how the dengue virus responds to the immune reactions of humans, and how people reach a state of protection against the four DENV serotypes, both at the level of antibodies (i.e., correlates of protection) and at the level of cellular memory. |
|  | Investigating vaccines | The development of effective antiviral agents and vaccines is a research gap that needs to be addressed. In this regard, new clinical trials and molecular studies need to be monitored for vaccine research. |

**Table E**. Perceived research themes in the dengue research landscape in LAC.

| **Research themes** | **Description** |
| --- | --- |
| Sociocultural research | Cultural aspects (e.g., gender norms, behaviours) must be addressed in social sciences research on dengue. There are also very few studies on the impact, or the absence of impact, of health actions at the social level (i.e., education, diagnosis and testing, vector control, etc.). Studies should address all these issues in an integrated way. The articulation between local studies and their regional context (i.e., climatic and economic) is essential. For this to work, social data must be geocoded and updated regularly in information systems and databases. |
| Entomological, viral, and immunological research | Addressing the biological factors through experimentation with local mosquito lineages may aid in understanding vectorial competence and capacity, and serotypes distribution. This entomoviral surveillance research must be conducted in a universal way. Additionally, what defines the epidemiological waves of DENV epidemics is the immunity of the population to the different serotypes. Therefore, research must also address human immunological traits as a biological factor in the eco-bio-social dengue episystem. |
| Vaccine development research | Although the existence of serotypes is a complication, the slow progress of vaccine development for DENV serotypes, compared to the development of vaccines against other viruses, where there are also variants, is striking. Therefore, research should investigate the clinical efficacy of potential vaccine candidates. |
| Epidemiological risk modeling | Research should consider remote epidemiological risk models and indicators, effective early warning systems, and preventive models that measure DENV risk in real time to inform early dengue diagnosis and prevention and control campaigns with clear impact measures. |

**Table F.** Summary of questionnaire items to evaluate the policy and program agenda for dengue prevention and control in LAC^a^.

| **Category** | **Item** | **Description** |
| --- | --- | --- |
| **Applicability** | Item 2.1 | The presented policy and program agenda can be applied to countries and territories of Latin America and Caribbean. |
|  | Item 2.2 | The presented policy and program agenda can be useful to governments and organizations in Latin America and the Caribbean. |
|  | Item 2.3 | The presented policy and program agenda can improve dengue prevention and control in Latin America and the Caribbean. |
|  | Item 2.4 | The presented policy and program agenda can be equitably implemented in Latin America and the Caribbean. |
|  | Item 2.5 | The presented policy and program agenda can be sustainability implemented in Latin America and the Caribbean. |
| **Generalizability** | Item 2.6 | The presented policy and program agenda can be generalizable to many or all countries and territories in Latin America and the Caribbean. |
|  | Item 2.7 | The presented policy and program agenda can be generalizable for other *Aedes*-transmissible diseases in Latin America and the Caribbean. |
| **Feasibility** | Item 2.8 | The presented policy and program agenda can be politically feasible in Latin America and the Caribbean. |
|  | Item 2.9 | The presented policy and program agenda can be technically feasible in Latin America and the Caribbean. |
|  | Item 2.10 | The presented policy and program agenda can be administratively feasible in Latin America and the Caribbean. |
| **Acceptability** | Item 2.11 | The presented policy and program agenda can be socially acceptable by communities in Latin America and the Caribbean. |
|  | Item 2.12 | The presented policy and program agenda can be politically acceptable by communities in Latin America and the Caribbean. |

^a^ 7-point Likert scale: strongly disagree; disagree; somewhat disagree; undecided; somewhat agree; agree; strongly agree.
